# Mild Cognitive Impairment as an Early Landmark in Huntington's Disease

**DOI:** 10.3389/fneur.2021.678652

**Published:** 2021-07-07

**Authors:** Ying Zhang, Junyi Zhou, Carissa R. Gehl, Jeffrey D. Long, Hans Johnson, Vincent A. Magnotta, Daniel Sewell, Kathleen Shannon, Jane S. Paulsen

**Affiliations:** ^1^Department of Biostatistics, College of Public Health, University of Nebraska Medical Center, Omaha, NE, United States; ^2^Department of Biostatistics, Indiana University Fairbanks School of Public Health, Indianapolis, IN, United States; ^3^Department of Psychiatry, College of Medicine, University of Iowa, Iowa City, IA, United States; ^4^Department of Biostatistics, College of Public Health, University of Iowa, Iowa City, IA, United States; ^5^Department of Electrical and Computer Engineering, University of Iowa, Iowa City, IA, United States; ^6^Department of Radiology, College of Medicine, University of Iowa, Iowa, City, IA, United States; ^7^Department of Neurology, University of Wisconsin, Madison, WI, United States

**Keywords:** prognosis, clinical trials, observational study, all cognitive disorders, dementia, mild cognitive impairment, Huntington's disease

## Abstract

As one of the clinical triad in Huntington's disease (HD), cognitive impairment has not been widely accepted as a disease stage indicator in HD literature. This work aims to study cognitive impairment thoroughly for prodromal HD individuals with the data from a 12-year observational study to determine whether Mild Cognitive Impairment (MCI) in HD gene-mutation carriers is a defensible indicator of early disease. Prodromal HD gene-mutation carriers evaluated annually at one of 32 worldwide sites from September 2002 to April 2014 were evaluated for MCI in six cognitive domains. Linear mixed-effects models were used to determine age-, education-, and retest-adjusted cut-off values in cognitive assessment for MCI, and then the concurrent and predictive validity of MCI was assessed. Accelerated failure time (AFT) models were used to determine the timing of MCI (single-, two-, and multiple-domain), and dementia, which was defined as MCI plus functional loss. Seven hundred and sixty-eight prodromal HD participants had completed all six cognitive tasks, had MRI, and underwent longitudinal assessments. Over half (i.e., 54%) of the participants had MCI at study entry, and half of these had single-domain MCI. Compared to participants with intact cognitive performances, prodromal HD with MCI had higher genetic burden, worsened motor impairment, greater brain atrophy, and a higher likelihood of estimated HD onset. Prospective longitudinal study of those without MCI at baseline showed that 48% had MCI in subsequent visits and data visualization suggested that single-domain MCI, two-domain MCI, and dementia represent appropriate cognitive impairment staging for HD gene-mutation carriers. Findings suggest that MCI represents an early landmark of HD and may be a sensitive enrichment variable or endpoint for prodromal clinical trials of disease modifying therapeutics.

## Introduction

It is well-known that Huntington's disease (HD) manifests as a triad of clinical symptoms (motor, cognitive, psychiatric); however, its diagnosis continues to rely primarily on the presence of motor impairment (1). Along with a paradigm shift in other movement disorders to examine non-motor components, cognitive impairment prior to HD motor diagnosis is now widely reported (2–5). Moreover, nearly two decades of research in persons with the gene expansion for HD have documented measures of earlier disease, but none are yet accepted as endpoints for preventive clinical trials. Patients with diagnosed or manifest HD are currently undergoing Phases II and III clinical trials for gene silencing using antisense oligonucleotides (6). Phase I clinical trials targeting somatic expansion have been announced, UniQure is testing a adeno-associated virus (AAV5) vector carrying an artificial micro-RNA specifically tailored to silence the huntingtin gene and several investigators are focusing on the genome-editing technology CRISPR. This work motivates the need for additional endpoints for clinical trials in early HD.

Mild cognitive impairment (MCI) has been used to describe individuals who experience cognitive difficulties greater than expected for their age but who fall short of a diagnosis of dementia. It has generally been reported that individuals with MCI progress to dementia more rapidly than their cognitively intact peers (7–10). Despite the evidence for cognitive deterioration in HD, MCI has largely been unused as a descriptor in the HD literature. It is well-known that individuals with MCI progress to develop a wide range of diagnostic conditions (Alzheimer's disease, vascular dementia, dementia with Lewy bodies, etc.) suggesting its applicability to a wide range of neurodegenerative disease. Increasingly, MCI has been recognized as an important diagnostic consideration in other movement disorders, such as Parkinson's disease (11, 12). MCI has been commonly determined by the age- and education-adjusted cut-off values in neurocognitive tests that are 1.5 standard deviations below the mean of its control peers (7, 10). One prior study (5) looked at applying the MCI criteria in prodromal HD finding that nearly 40% of their sample of individuals with HD met criteria for MCI. A recent study (13) also investigated MCI in motor-manifest HD individuals and reported the rate of MCI as high as 90% in this cohort according to the criteria.

MCI has been utilized as a means to identify individuals at increased risk of developing neurodegenerative disease (i.e., Alzheimer's disease) to enroll in formal clinical trials (14). However, heterogeneity among those with the MCI diagnosis has presented a challenge to these clinical trials (15). As HD is caused by expansion of the trinucleotide cytosine-adenine-guanine (CAG) in the *HTT* gene (16), there is the ability to identify individuals who will develop HD prior to symptomatic presentation of the disease. Validation of earlier signs of the disease may be timely to facilitate preventive clinical trials prior to motor diagnosis.

Our analysis relies on PREDICT-HD, which is an observational study with a large cohort of individuals with the gene mutation for HD who were clinically determined to be free of motor diagnosis at study entry based on traditional motor criteria. There are two main goals of this study: the first is to classify the gene-expanded individuals as either having MCI or not based on the controls (not gene-expanded) and to examine the extent to which the classification is valid; the second goal is to estimate the timing of MCI and construct a model for the evolution of cognitive impairment over time, from cognitively-intact to MCI to dementia. The results will be informative for evaluating the extent to which MCI in HD gene-mutation carriers is a defensible indicator of early disease potentially appropriate as an endpoint in preventive trials.

## Materials and Methods

### Study Design and Participants

The PREDICT-HD study was a 12-year prospective observational study at 32 sites in six countries (USA, Canada, Germany, Australia, Spain, and UK) from September, 2002 to April, 2014. The study recruited a total of 1,155 HD gene-expanded individuals and 317 controls (not gene-expanded) individuals who were mainly family members of the gene-expanded individuals. The gene-expanded individuals (CAG length >35) previously underwent independent genetic testing for the HD gene-expansions, and had diagnostic confidence level (DCL) ratings of the Unified Huntington's Disease Rating Scale (UHDRS) <4 at the study entry. Ancillary studies supported continuation of this research at a reduced number of sites (*n* = 8) through 2017. All individuals enrolled in PREDICT-HD had independently undergone predictive testing for HD and know their genetic finding. All study participants underwent annual evaluation.

### Standard Protocol Approvals, Registrations, and Patient Consents

Ethical standards were reviewed at the primary grant institution and all participating sites. All participants signed a written informed consent allowing data sharing for future research. The multi-site research study is identified on ClinicalTrials.gov Identifier: NCT00051324, Neurobiological Predictors of Huntington's Disease (PREDICT-HD) and is shared in dbGaP.

### Study Variables

#### Cognition

Since cognitive assessment was comprehensive in the PREDICT-HD study, we used previous findings to choose a smaller discrete set of cognitive tasks. Harrington et al. (17) conducted a factor analysis of 18 tests to identify latent factors that elucidated core cognitive constructs for prodromal HD. Findings showed six cognitive factors in prodromal HD including inhibition, working memory, motor planning, information integration, sensory processing, and learning memory (17). For each cognitive factor, we chose the one test outcome with the largest sample size to represent each cognitive domain: the Stroop Color Word Test (STROOP) (18) for inhibition, WAIS-III Letter Number Sequencing (LNS) (19) for working memory, paced tapping (PACE) (20) for motor planning, Symbol Digit Modalities Test (SDMT) (21) for information integration, smell identification test (SMELL) (22) for sensory processing and the Hopkins Verbal Learning Test free recall (HVLT) (23) for learning with total observations of 7,492, 4,203, 2,547, 7,503, 4,556, and 2,505, respectively. The large variation in cognitive task frequencies is because the UHDRS cognitive assessments were administered every year whereas all other cognitive tasks were administered every other year to maximize the number of tasks piloted for sensitivity in this first-in-human multi-site prodromal HD study. Cognitive tasks were started and stopped during the 12-year study based on feasibility, psychometric principles (reliability, construct validity) participant burden, and effect size differences from controls at baseline and change over time. There were 768 prodromal HD individuals who had completed all selected cognitive measures, had MRI and longitudinal assessment. Comparison of these cognitive outcomes with other research showed that three (of four) were used in our previous MCI cross-sectional paper (5), four (of five) were used in our paper showing the best cognitive tasks for HD neuroanatomical associations using MRI (24), and all six cognitive outcomes were used in a study to examine the greatest changes over time in prodromal HD (4). These six cognitive tasks were selected for the current study to examine the clinical utility of a brief battery for clinical care and MCI diagnostics.

**MRI acquisitions** were collected using high resolution anatomical MR images at 32 collection sites (53 unique scanners) using General Electric, Phillips, and Siemens scanners with field strengths of 1.5 T (Tesla) or 3 T. T1 images at each site were obtained using three-dimensional (3D) T1-weighted inversion recovery turboflash (MP-RAGE) sequences. Each imaging data set was processed through data processing pipelines optimized for data harmonization across multiple sites (BAW-REF) (25). The fully automated processing includes automated landmark detection (BCD), bias field correction (BABC, BABC) (26, 27), and multi-atlas label fusion (MALF) (28).

**Genetic burden** was defined using the CAG repeat length by age product, or CAP (29) score, where the CAG repeat length is scaled and then multiplied by age at entry (Age_0_) for each individual research participant, i.e., CAP = Age_0_ × (CAG-−33.66). Thus, individuals with higher genetic burden/CAP scores are statistically nearer to motor diagnosis (29).

**Severity of motor impairments** was defined using the sum of the 31-item Total Motor Score (TMS) from the UHDRS (30, 31). The TMS is the sum of motor abnormalities observed during a standardized neurological examination and ranges from 0 to 124.

#### HD Diagnosis

A research diagnosis of HD was given by a certified motor rater trained by the Huntington Study Group (HSG) who scored each research participant on the UHDRS Diagnostic Confidence Level (DCL) (30). DCL ranges from 0 = having no motor abnormalities to 4 = >99% confident that motor abnormalities are definitive signs of HD, and DCL = 4 is the definition of motor diagnosis.

**Total Functional Capacity** (TFC) is a type of activities of daily living scale from the UHDRS. Specifically, the TFC is a clinician-rated measure of functional capacity based on a standardized interview with the participant and available family members of current independent functioning in these domains: finances, driving, living independently, bathing, feeding, and walking. The scale ranges from 1 to 13 and any loss in independence is considered abnormal for persons without disability.

### Statistical Methods

The analysis consisted of three steps, the first being the classification of gene-expanded individuals as MCI performed at each visit, the second step was to use MCI status at baseline to predict the timing of HD onset, the third step was to demarcate stages in longitudinal cognitive decline. For the first step, a control-referenced (or norm-referenced) approach to MCI classification was used, which is in the same spirit as in the general approach often used in Alzheimer's (32), Parkinson's (33), and Huntington's disease (5, 13), but with a more advanced modeling technique to accommodate the longitudinally-collected neurocognitive test information. For each of the six cognitive variables, a linear mixed-effects model (LMEM) was fitted for the PREDICT-HD controls, adjusted for age, education years, and number of the same test that had been done prior, to obtain the age-, education-, practice effect-adjusted prognostic distribution of test values. The fitted control distribution of these test values was treated as the reference distribution for classifying gene-expanded individuals as MCI. At each visit, a gene-expanded individual was classified as MCI if their score was worse than or equal to 1.5 standard deviations below the mean of the control individuals adjusted for age, education level and practice effect. The age that the gene-expanded individual's test value crossed the adjusted cut-off the first time was defined as the age at detected MCI for that individual and for the domain represented by the cognitive variable. The detailed model derivation for the cut-off of MCI classification can be found in the Web Supporting Materials.

As a check on the validity of the classification, for each cognitive domain several baseline variables were compared between individuals with MCI and those who were cognitively intact. Comparisons of baseline variables were conducted using *t*-test for continuous variables and chi-square tests for categorical variables. At study entry, brain atrophies for prodromal HD participants in terms of percentage loss of brain volume were compared to the mean of control individuals. Several structures were considered, including caudate, putamen, globus pallidus, total gray matter, total white matter, and 95% confidence intervals were estimated. For all the comparisons, the significance level was set at 0.05.

The second step in the analysis was to formulate a prediction model for examining the ability of MCI status at baseline to predict the timing of the HD onset indicated by motor diagnosis, adjusting for covariates. All 1,155 gene-expanded individuals were used for the analysis. MCI status at study entry was coded into one of three impairment levels: 0-No; 1-Yes; 2-unknown, with the latter being due to missing observations. Flexible accelerated failure time (AFT) models with interval-censored (34) observations for HD onset were used, with adjustment variables being CAP and TMS at study entry. Interval-censoring was assumed because PREDICT-HD has annual visits and the exact time of HD onset cannot be exactly determined (e.g., conversion could happen mid-way between two visits). Consistent with best practice, several parametric forms of the AFT were fitted, and the log-logistic AFT model (29) was found to be optimal based on Akaike's Information Criterion (AIC). The predicted median onset time of HD in years since study entry and its 95% confidence interval (CI) for any given CAP and TMS values were calculated based on the log-logistic AFT model.

The third and final step was to stage the cognitive impairment for each prodromal HD individual, based on the following taxonomy assumed to be ordered in time. Cognitively-intact: no evidence of MCI or other cognitive impairment; MCI-Single: MCI classification in only one cognitive domain; MCI-Duo: MCI classification in two cognitive domains; MCI-Multiple: MCI classification in three or more cognitive domains; dementia (DM): HD-specific dementia defined as MCI classification in any domain with at least a one-point loss on the TFC. For each interval-censored stage of cognitive impairment (left-censored if the stage was diagnosed at the first observation, interval-censored if the stage was determined in adjacent times during the follow-up, and right-censored if the stage was not able to classify at the last observation), we fitted a Weibull AFT model for the onset age using both CAG and CAG (2) as predictors because this AFT model was found to be optimal based on the AIC. We then calculated the CAG-specific median onset age and its 95% CI to ascertain the time gap between the median onset ages among the stages of cognitive impairment.

## Data Availability Policy

All data, including raw and processed images, are provided in dbGaP PREDICT-HD Huntington's Disease Study: ninds-dac@mail.nih.gov.

## Results

In the PREDICT-HD study, the HD gene-expanded individuals had a mean age 39.9 years (SD 10.6; range 18.1–75.9), a mean education year 14.5 years (SD 2.6; range 8–20), 64.4% were women, and 97.1% were white. The control individuals were statistically a little older (*p* <0.0001) than the HD gene-expanded individuals with a mean age 44.1 years (SD 12.1; range 19.2–83.7) and received comparable education with a mean education year 14.9 years (SD 2.4; range 9–20), though statistically different at level 0.05 (*p* = 0.012) due to large sample size. 64.8% and 98.9% of the control individuals were women and white, respectively, which were not statistically different from the gene-expanded individuals at level 0.05.

Table 1 shows the nature and frequency of the MCI classification at baseline. Among all 768 gene-mutation carriers who had complete cognitive assessment data, 411 (54%) showed MCI at baseline and 357 were cognitively intact (46%). 207 (27%) were classified with MCI in a single domain, 105 had MCI in two domains, and 99 had MCI in three or more domains.

**Table 1:** The frequency of MCI patterns at study entry (baseline).

**MCI patterns**	**Frequency**
**Cognitively intact**	357
**MCI in single domain**	
Inhibition	15
Working memory	18
Motor planning	63
Information integration	36
Sensory processing	35
Learning—memory	40
Subtotal	207
**MCI in two domains**	
Inhibition and motor planning	6
Inhibition and Information integration	7
Inhibition and sensory processing	1
Inhibition and verbal learning—memory	2
Working memory and motor planning	2
Working memory and information integration	6
Working memory and sensory processing	1
Working memory and learning—memory	6
Motor planning and information integration	21
Motor planning and sensory processing	14
Motor planning and learning—memory	11
Information integration and sensory processing	4
Information integration and learning—memory	16
Sensory processing and learning—memory	8
**Subtotal**	105
**MCI in at least three domains**	99
**Total**	768

*MCI, Mild Cognitive Impairment*.

Table 2 provides clinical and demographic data at study entry comparing gene-expanded individuals who were classified as MCI vs. those who were not (cognitively intact or pre-MCI). Individuals classified as MCI in any cognitive domain had significantly higher genetic burden (CAP) and worse motor impairment (TMS) at baseline. The most prevalent MCI was observed in information integration (31%), followed by learning (28%), and then motor planning (25%); the remaining MCIs were observed in <19% of the sample.

**Table 2:** Summary statistics of clinical and demographic variables among MCI groups at study entry (baseline).

	**Cognitively intact**	**MCI- inhibition**	**MCI-working memory**	**MCI-motor planning**	**MCI-information integration**	**MCI-sensory processing**	**MCI- learning—memory**
*N* (% of total prodromal HD sample)	357 (47%)	130 (17%)	94 (12%)	195 (25%)	240 (31%)	145 (19%)	214 (28%)
**Continuous variables**							
**Age**							
Mean	40.3	38.7	41.3	40.8	38.6^*^	42.5^*^	40.3
SD	9.4	9.9	10.0	9.8	10.4	11.1	10.0
Range	[21.8, 72.9]	[20.3, 65.7]	[26.0, 65.6]	[18.1, 75.9]	[20.0, 67.9]	[18.1, 75.9]	[23.2, 75.0]
**CAP**							
Mean	316.4	379.9^***^	392.6^**^	390.9^**^	386.0^**^	391.0^**^	382.1^***^
SD	70.7	98.7	82.7	87.4	92.8	88.1	90.4
Range	[145.7, 505.5]	[168.1, 845.8]	[186.2, 652.0]	[168.1, 845.8]	[111.1, 845.8]	[119.0, 652.0]	[160.3, 845.8]
**TMS**							
Mean	3.3	10.1^**^	8.6^**^	7.6^**^	8.8^**^	7.9^**^	8.0^***^
SD	3.5	9.7	9.1	6.4	8.7	9.2	7.9
Range	[0, 18]	[0, 47]	[0, 44]	[0, 34]	[0, 47]	[0, 47]	[0, 44]
**Categorical variable** ***n*** **(%)**							
**Sex**							
Female	232 (65.0)	80 (61.5)	57 (60.5)	152 (78.5)^**^	139 (57.9)^**^	66 (45.5)^**^	100 (44.6)^**^
Male	125 (35.0)	50 (38.5)	37 (39.5)	43 (21.5)	101 (42.1)	79 (54.5)	113 (55.4)
**Race**							
White	347 (97.2)	126 (96.9)	94 (100)	192 (98.5)	236 (98.3)	139 (95.9)	207 (96.7)
Others	10 (2.8)	4 (3.1)	0 (0)	3 (1.5)	4 (1.7)	6 (4.1)	7 (3.3)

*MCI, Mild Cognitive Impairment; CAP, CAG by Age Product; TMS, Total Motor Score from the Unified HD Rating Scale*.

*** * p < 0.001*;

** *0.001 ≤*

*p < 0.01*;

* *0.01 ≤*

*p < 0.05*.

Table 3 shows volumes and average percentage brain loss at baseline compared to the mean of control individuals (with 95% confidence intervals) for the six brain regions. Except for cerebral gray matter, MCI individuals showed significant brain atrophy in caudate, putamen, globus pallidus, and cerebral white matter at study entry relative to controls. Particularly in the regions of caudate, putamen and globus pallidus, the cognitively intact (pre-MCI) group showed 10% or more loss in brain volume on average in reference to the mean volume of controls. For individuals with any MCI diagnosis at study entry, brain atrophy was >20% loss, on average.

**Table 3:** Summary of average percentage brain atrophy (and its 95% confidence interval) in the six selected brain regions for prodromal HD individuals with and without MCI.

**MRI volumes**	**Control group mean**	**Cognitively intact**	**MCI inhibition**	**MCI working memory**	**MCI motor planning**	**MCI information integration**	**MCI sensory processing**	**MCI learning memory**
Caudate	6590.5	−9.9 (−12.3, −7.6)	−20.6 (−24.7, −16.5)	−24.5 (−28.8, −20.3)	−25.4 (−28.3, −22.4)	−22.5 (−25.4, −19.6)	−21.2 (−24.9, −17.5)	−18.5 (−21.6, −15.5)
Putamen	8554.2	−11.2 (−13.4, −8.9)	−21.7 (−25.8, −17.6)	−24.5 (−28.5, −20.5)	−27.1 (−30.0, −24.2)	−23.2 (−26.1, −20.3)	−22.9 (−26.6, −19.2)	−18.6 (−21.7, −15.5)
Globus Pallidum	2765.3	−12.8 (−15.0, −10.5)	−22.2 (−26.6, −17.7)	−26.5 (−30.7, −22.2)	−29.0 (−32.0, −26.0)	−23.9 (−26.9, −20.9)	−23.2 (−27.0, −19.2)	−18.8 (−22.1, −15.5)
Cerebral Gray Matter	112116.6	0.3 (−1.4, 2.1)	−0.4 (−2.8, 2.0)	0.3 (−2.0, 2.7)	−1.1 (−3.0, 1.0)	0.6 (−1.4, 2.6)	2.6 (0.2, 5.0)	1.8 (−0.2, 3.9)
Cerebral White Matter	26074.3	−3.8 (−6.0, −1.5)	−4.5 (−7.6, −1.3)	−5.5 (−9.2, −1.4)	−8.4 (−10.7, −6.0)	−4.5 (−7.1, −1.8)	−2.8 (−5.9, 0.4)	−3.1 (−5.8, −0.4)

*MRI, Magnetic Resonance Imaging; MCI, Mild Cognitive Impairment*.

*Values are mean differences in percentage relative to the control group (negative indicates less than control mean)*.

Supplementary Table A shows the results of the log-logistic AFT model for time to HD onset from baseline, using MCI classification, CAP, and TMS at baseline as predictors. The motor planning MCI (*p* = 0.014) had a statistically significant effect and contributed to prediction of HD onset above and beyond genetic burden (CAP) and motor abnormality (TMS).

For the prodromal HD individuals who were cognitively intact at baseline, 343 had follow-up observations for cognitive assessments. Among them, 48% were classified for having MCI during follow-up: 92 showed single domain MCI, 31 showed MCI in two domains and 40 had MCI in three or more cognitive domains (see Supplementary Table B).

**Figure 1:**
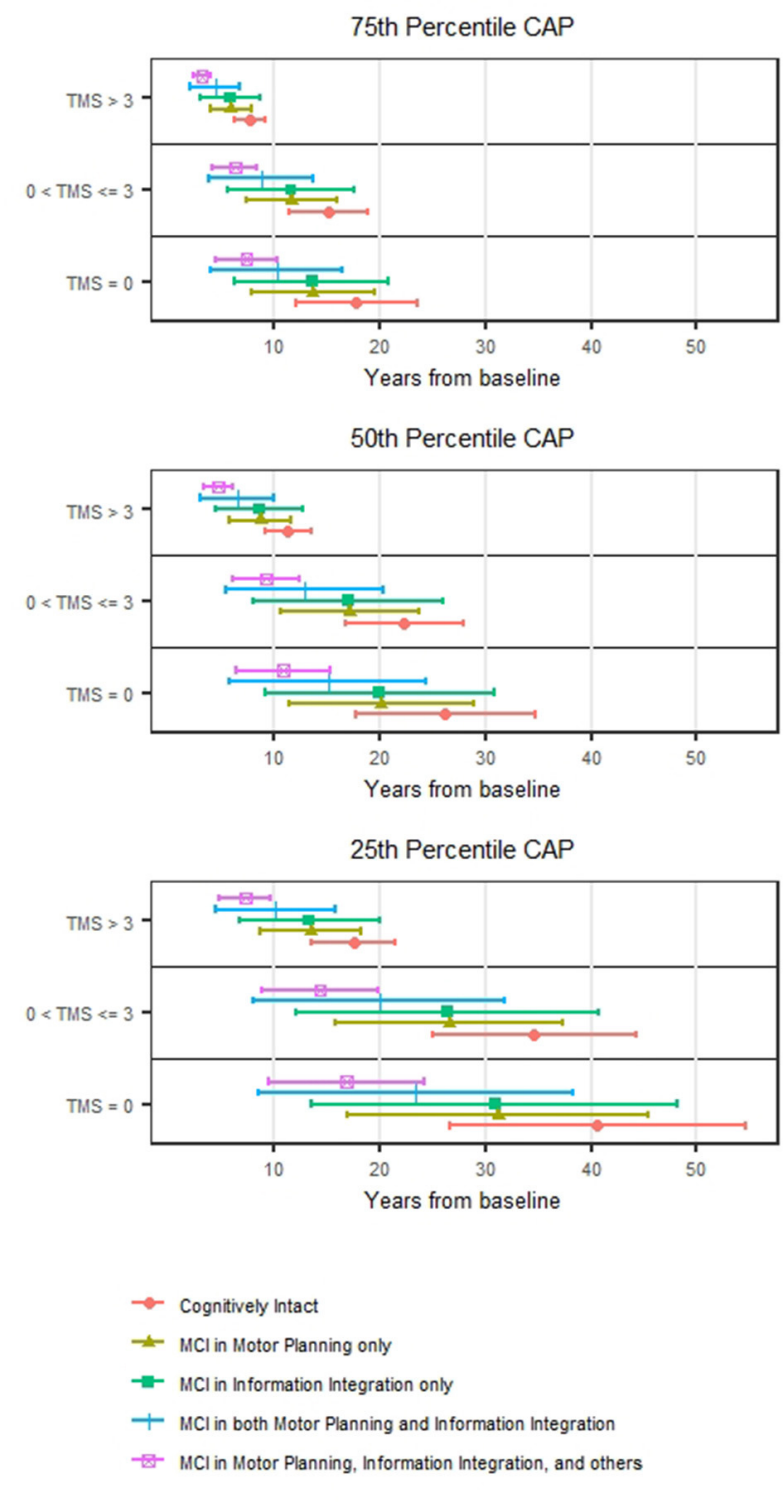
Prediction of HD motor onset. The estimated median motor onset time from the baseline and its 95% confidence interval stratified by TMS (0, >0–3, and >3) for individuals with CAP at the 25th, 50th, and 75th percentiles.

Figure 1 shows the estimated median time to HD onset from baseline and its 95% confidence interval (CI) at selected values of CAP (the 25th percentile = 279.6; 50th percentile = 340.5; and 75th percentile = 394.2) and TMS (0; 1–3; >3) and TMS classification made by 0 (no motor impairment) and 3 (median value of motor impairment at study entry) by MCI group. The figure indicates that the median time tends to be shorter as TMS increases and MCI group increases in severity. For example, for those with CAP at the 75th percentile and TMS >3 at study entry, the median time to HD onset from baseline was estimated at 7.7 years (95% CI 6.2–9.2) for cognitively intact (pre-MCI), and 3.2 years (95% CI 2.3–4.0) for MCI in motor planning, information integration and others at the study entry. The non-overlapping of the 95% CIs indicates a statistically significant difference in median HD onset times between the two groups that only differ in MCI severity.

**Figure 2:**
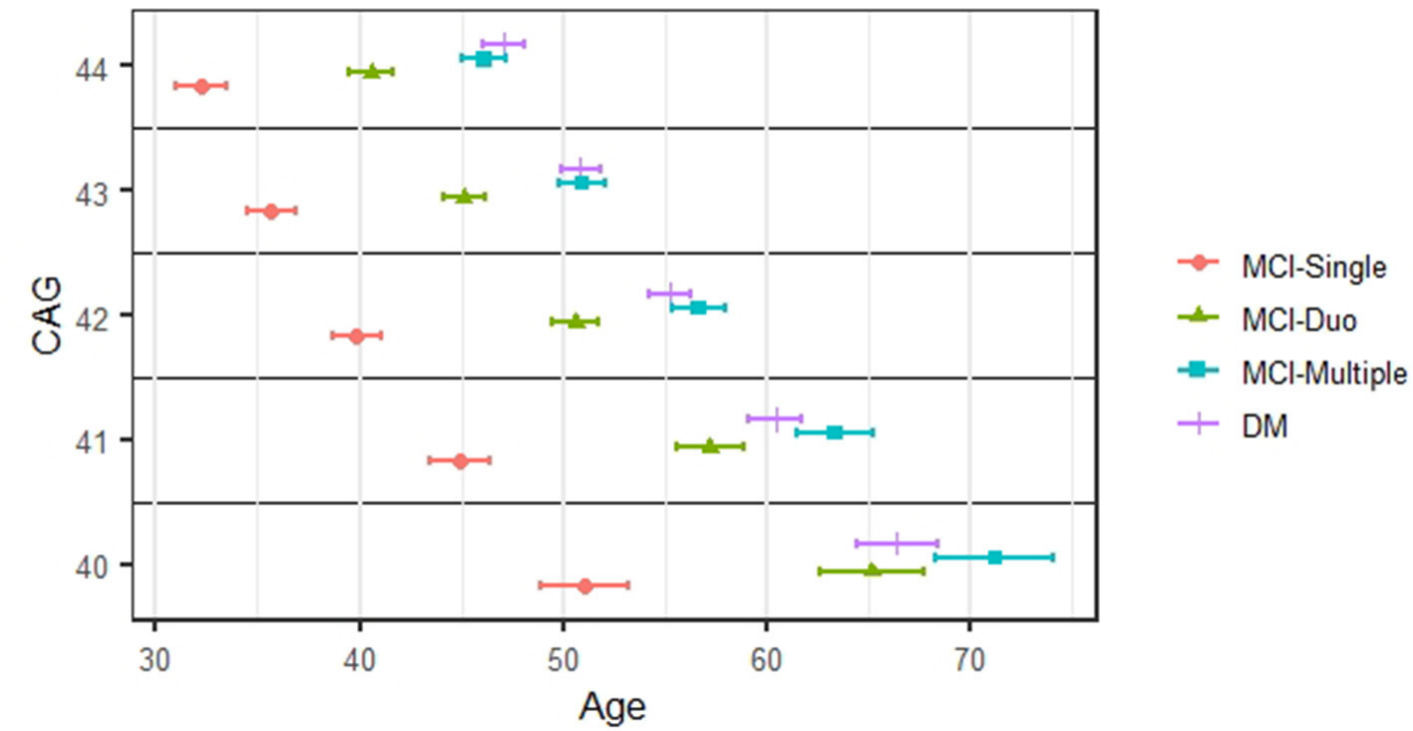
Staging of cognitive impairment. The estimated median onset age and its 95% confidence interval for each stage of cognitive impairments.

Figure 2 shows the staging results in the form of the estimated median age at onset for each stage of cognitive impairment (and its 95% CI). For brevity, we present results for CAG values between 40 and 44, based on the Weibull AFT model. Within any CAG stratum, the three MCI stages are quite separated. For example, for prodromal HD individuals with CAG values of 40, the median onset age was 51.1 years for MCI-Single (95% CI 48.9–53.2), 65.2 for MCI-Duo (95% CI 62.6–67.7), and 71.2 for MCI-Multiple (95% CI 68.3–4.1). There was much less separation for dementia (DM) and MCI-Multiple, with the CIs for each always overlapping.

## Discussion

Cognitive impairment has long been documented among individuals with prodromal HD (2, 4, 35, 36). Recently, a task force in the Movement Disorder Society has been formed to propose new classifications of HD that considered cognitive impairment in addition to motor disorder, and called for longitudinal ascertainment of cognitive disorder (37). As an early index event for cognitive impairment, MCI was originally defined and widely accepted in Alzheimer's disease (32), and extended to Parkinson's disease (33) and Huntington's disease (5, 13) though the concept of MCI has been controversial for Parkinson's and Huntington's diseases (38). Following the criteria of HD specific MCI (5, 13), we developed the sophisticated statistical models to identify age at MCI onset for various cognitive domains for HD gene-expanded individuals by leveraging PREDICT-HD 12-year follow-up data on neurocognitive assessments, which helped shed light on classification of early HD (37).

As a whole, our results provide biological and prognostic validity for MCI in prodromal HD. For our sample, MCI was prevalent at baseline, affecting more than half the sample. Furthermore, for those who did not have MCI at baseline, about half were classified as MCI over time. Longitudinal MCI trends suggest that gene-expanded individuals start with single-domain MCI, then develop multiple-domain MCI and eventually dementia.

The supporting evidence for the MCI classification at baseline is compelling. Compared with cognitively intact prodromal HD individuals at study entry, brain atrophy was significantly worse among those who showed MCI in any of six domains. The results of brain atrophy reference to the control group shown in Table 3 were conservative since the control cohort was a little older than the gene-expanded cohort in the PREDICT-HD study. The atrophy would be more significant should age be adjusted. Additionally, MCI was a clinically relevant prognostic marker for HD motor onset; prodromal HD individuals with MCI at study entry had earlier HD motor onset than those who were cognitively intact at study entry. Notably, for prodromal HD individuals who were classified as MCI in motor planning, brain atrophy in the basal ganglia was most severe. Prognostic validity for MCI in HD remained after adjusting for known predictors of HD onset (i.e., CAP and TMS), prodromal HD individuals with motor planning MCI at study entry had significantly elevated risk for earlier manifestation of HD motor onset when compared with their counterparts without motor planning MCI.

These findings have clinical and research implications for individuals at HD risk. First, our results support the viewpoint that MCI should be recognized as a clinically important early landmark event in the course of HD parallel to its importance in both Alzheimer's and Parkinson's diseases. Early clinical identification of MCI can facilitate future planning for individuals with HD and their families. Second, while MCI has largely been used as a means for identifying a population to enroll in clinical trials for Alzheimer's disease, the high prevalence of MCI among prodromal HD individuals and the significant biological and prognostic validity for HD diagnosis suggest that MCI has potential value as a study endpoint in clinical trials for disease modification. Finally, MCI may prove an enrichment strategy for the design of clinical trials. For example, utilizing the presence or absence of MCI within a cohort of individuals with prodromal HD could seek to reduce heterogeneity within a prodromal sample and further clarify the probable nearness to clinical onset of motor symptoms and thus formal diagnosis of HD.

Findings from this study document that 54% of the sample met criteria for MCI at study entrance, which is higher than previously published rates (5). This increase is not surprising given that the current study used all six cognition domains (vs. their use of 4 cognitive domains) identified in a large factor analysis of premanifest HD participants (17). The MCI literature emphasizes that breadth and depth of cognitive domains selected for MCI consideration can impact its detection. A research study combining the Framingham Heart Study (*n* = 915) with the Mayo Clinic Study of Aging (*n* = 1,969) suggested that though dementia incidence rates were similar across studies, heterogeneity was observed in hazard ratios and positive predictive values across cognitive domains (39). The authors concluded that the popularly-used cutoff score of−1.5 SD below the mean “represents a reasonable compromise for making the categorical diagnosis of MCI clinically meaningful (p. 1718),” though risk of future dementia is logically related to the distinct depth and breadth of cognitive assessments conducted and related depth and breadth of impairments found. The current study builds upon the earlier MCI HD study in that it provides additional documentation regarding the relationship between the classification of MCI in prodromal HD and other indicators of disease burden including basal ganglia atrophy and years to motor onset. Moreover, longitudinal assessment of MCI classification is offered. Altogether, this provides additional support for the validity of the use of MCI in prodromal HD.

Numerous clinical trials have implemented the use of MCI as a means of selecting individuals for clinical trials in Alzheimer's disease (14, 40–43). Unfortunately, to date, all have failed to show efficacy of any targeted interventions. Petersen and colleagues (15) speak eloquently regarding the probable impact of significant heterogeneity in the MCI samples contributing to these findings. In addition, they speak about the importance of consideration of endpoint measures noting that for some MCI samples with mild deficits, dementia may be too distant of an endpoint. Ultimately, they call for combined use of the MCI diagnosis and other biomarkers to stratify and thus reduce heterogeneity in the sample.

In the consideration of clinical trials for individuals with prodromal HD, these difficulties encountered in the MCI clinical trials must be considered. The present findings are encouraging with regard to these possible prodromal HD clinical trials. First, the use of MCI as a selection tool may help to reduce heterogeneity within the prodromal sample. That is individuals with prodromal HD and MCI would reflect a group at high risk for development of the motor diagnosis of HD, particularly if used in conjunction with information regarding their CAG repeat length and age. Additionally, unlike the MCI clinical trials completed to date, the genetic nature of HD provides the opportunity to use MCI as a clinical endpoint for individuals with prodromal HD. Such a clinical trial may allow for clinical trials even earlier in the course of their disease, a goal for neurodegenerative disease clinical trials in general.

While this work constitutes a comprehensive study for MCI in prodromal HD individuals, it has limitations. Since individuals pursuing HD genetic testing, like those in this study, comprise a smaller proportion of individuals at-risk of HD (16) the PREDICT-HD sample may not represent the whole prodromal HD population. Because observations of neuropsychological tests in selected cognitive domains were not balanced in size at each study visit, replications are expected to reveal minor variations in proportions of each MCI domain, based on tasks and domains used. That is, some tests may detect fewer MCI observations in prodromal HD whereas other tasks may detect more. Further research is warranted to validate the most sensitive assessment strategy for the earliest detection and tracking of disease in HD. Though it might have been preferable to use the composite score for each of the factor score domains (17) for diagnoses of MCI, it is not possible due to the inconsistently administered cognitive assessments from PREDICT-HD and the subsequently reduced numbers of cognitive tasks being integrated into ongoing observational studies such as ENROLL. The definition of MCI in this paper did not consider any social cognition measures included in the *Diagnostic and Statistical Manual-Fifth Edition* (44) as they did not meet our sample size criteria for inclusion. Measures of social cognition in PREDICT-HD have been reported elsewhere (e.g., emotion recognition) and are likely to increase the frequencies of MCI in HD when included. It is also important to note that a high proportion of HD gene-mutation carriers display significant anosognosia through the course of HD (35), which may be highly correlated with cognitive impairment (13, 45). However, PREDICT-HD did not contain measures for subjective cognitive complaint and therefore did not allow for assessing the potential impact of anosognosia on MCI in HD. The HD community must await an appointed task force for consensus definition of HD-specific MCI for more widespread adoption in clinical and research practices.

Despite these limitations, this work provides a vivid description of cognitive change in prodromal HD and increased validation of the use of MCI in this sample. MCI occurs in over half of persons at genetic risk for HD, is associated with brain atrophy of the basal ganglia, and has prognostic validity for age at motor onset and dementia. Such information may help researchers and clinicians alike better understand and identify cognitive progression in Huntington disease.

## Data Availability Statement

Publicly available datasets were analyzed in this study. This data can be found at: National Center for Biotechnology Information (NCBI) dbGaP, https://www.ncbi.nlm.nih.gov/gap/, PREDICT-HD Huntington Disease Study (phs000222.v6.p2).

## Ethics Statement

The studies involving human participants were reviewed and approved by Ethical standards were reviewed at the primary grant institution and all participating sites. All participants signed a written informed consent allowing data sharing for future research. The multi-site research study is identified on ClinicalTrials.gov Identifier: NCT00051324, Neurobiological Predictors of Huntington's Disease (PREDICT-HD). The patients/participants provided their written informed consent to participate in this study.

## Author Contributions

YZ and JP conceived and designed the study. YZ, JZ, and JP acquired, analyzed, or interpreted the data, and drafted the manuscript. CG, JL, HJ, VM, DS, and KS provided critical insights on intellectual content and important revision of the manuscript. YZ and JZ conducted statistical analysis. JP and YZ obtained funding to conduct this study. All authors contributed to the article and approved the submitted version.

## Conflict of Interest

The authors declare that the research was conducted in the absence of any commercial or financial relationships that could be construed as a potential conflict of interest.
