# Supplementary material for: Mild Cognitive Impairment as an Early Landmark in Huntington's Disease
Source: Front Neurol. 2021 Jul 7;12:678652. doi: 10.3389/fneur.2021.678652 (PMC8292715; doi:10.3389/fneur.2021.678652)
Supplement: Supplementary file 1 [file Data_Sheet_1.PDF]

Supplementary Material: MILD COGNITIVE IMPAIRMENT AS AN EARLY LANDMARK IN HUNTINGTON DISEASE

Classification of MCIs

The six neuropsychological test scores: Stroop Interference (STROOPIN), Dual Verbal Working Memory (VERFLCOR), Cued Movement Sequencing (TA2T_M), Symbol Digit Modalities Test (SDMT) and Correct Percentage in Smell Identification Task (SMELL_PCT) and Delayed Recall (HVLT_TOTLEARN), were periodically collected for both 1155 HD gene-expanded and 317 non-gene-expanded individuals in the PREDICT-HD study. The longitudinal observations on the six test scores from the 317 HD non-gene-expanded individuals were treated as normative data to establish the following linear mixed-effects models:

$$Y_{ij,k} = \alpha_{0,k} + \alpha_{1,k} \times Age_{ij} + \alpha_{2,k} \times Practice_{ij,k} + \alpha_{3,k} \times Education\_Year_i + a_{i,k} + \varepsilon_{ij,k}$$

In the model, $Y_{ij,k}$ stands for the kth neuropsychological test score observed at the jth follow-up visit for the ith HD non-gene-expanded individual with $k = 1, \cdots, 6$ representing STROOPIN, VERFLCOR, TA2T_M, SDMT, SMELL_PCT, and HVLT_TOTLEARN, respectively; $a_{i,k} \sim N(0, \delta_k^2)$ is the random-effect indicating the between-individual variation for the kth neuropsychological test score; $\varepsilon_{ij,k} \sim N(0, \sigma_k^2)$ is the independent random error indicating the within-individual variation for the kth neuropsychological test score. AGE_{ij} is the age of the ith HD non-gene-expended individual at the jth follow-up visit. $Practice_{ij,k}$ represents the number of tests that have been conducted for the kth neuropsychological test in the ith HD non-gene-expanded individual at the jth follow-up visit indicating the practice effect. $Education\_Year_i$ is the year of education that the ith HD non-gene-expanded individual had at the study entry.

The models were fitted using SAS 9.4 and the estimates of model parameters were summarized in the following table.

Table 1. The Estimates of the Model Parameters for the Six Neuropsychological Tests

Test	$\hat{\alpha}_0$	$\hat{\alpha}_1$	$\hat{\alpha}_2$	$\hat{\alpha}_3$	$\hat{\delta}$	$\hat{\sigma}$
**STROOPIN**	50.67	-0.277	0.699	0.528	8.69	4.31
**VERFLCOR**	37.82	0.059	0.204	0.499	10.42	5.56
**TA2T_M**	216.86	0.597	-0.292	-0.833	21.70	17.75
**SDMT**	60.53	-0.322	0.476	0.553	7.81	4.81
**SMELL_PCT**	90.86	-0.214	0.214	0.316	5.91	7.45
**HVLT_TOTLEARN**	26.21	-0.064	0.327	0.289	3.02	2.91

Based on the results in Table 1, the cut-off values for classifying MCIs adjusting for age, practice effect and year of education each of the six domains can be determined in the following manner. Suppose for HD gene-expended individuals with year of education of EDUCATION_YEAR who were evaluated at age

of AGE with the times of prior tests for the kth neuropsychological test, PRACTICE. The mean of normative data for the kth neuropsychological test score for this group is estimated by

$$\hat{\hat{\mu}}_k = X^T \hat{\alpha}_k = \hat{\alpha}_{0,k} + \hat{\alpha}_{1,k} \times \text{AGE} + \hat{\alpha}_{2,k} \times \text{PRACTICE} + \hat{\alpha}_{3,k} \times \text{EDUCATION\_YEAR}$$

with $X = (1, \text{AGE}, \text{PRACTICE}, \text{EDUCATION\_YEAR})^T$ and $\hat{\alpha}_k = \left(\hat{\alpha}_{0,k}, \hat{\alpha}_{1,k}, \hat{\alpha}_{2,k}, \hat{\alpha}_{3,k}\right)^T$. The variance of normative data for Age-Practice Effect-Education Year adjusted k^{th} neuropsychological test score the can be estimated by

$$\hat{V}_k = X^T \widehat{cov}(\hat{\alpha}_k) X + \hat{\delta}_k^2 + \hat{\sigma}_k^2$$

with the variance-covariance matrix of estimated regression parameters $\widehat{cov}(\hat{\alpha}_k)$ given in Table 2.

Table 2. The estimates of variance-covariance matrix of the estimated model parameter

	**STOOPIN**	**VERFLCOR**
	$\begin{pmatrix} 13.95 & -0.0801 & 0.0810 & -0.6808 \\ -0.0801 & 0.0020 & -0.0023 & -0.0005 \\ 0.0810 & -0.0023 & 0.0054 & 0.0006 \\ -0.6808 & -0.0005 & 0.0006 & 0.0471 \end{pmatrix}$	$\begin{pmatrix} 26.36 & -0.1735 & 0.1262 & -1.2355 \\ -0.1735 & 0.0044 & -0.0046 & -0.0010 \\ 0.1262 & -0.0046 & 0.0296 & 0.0009 \\ -1.2355 & -0.0010 & 0.0009 & 0.0867 \end{pmatrix}$
	**TA2T_M**	**SDMT**
**Estimated Var-Cov Matrix**	$\begin{pmatrix} 144.72 & -0.9592 & 0.3328 & -6.7100 \\ -0.9592 & 0.0246 & -0.0263 & -0.0061 \\ 0.3328 & -0.0263 & 0.3481 & 0.0045 \\ -6.7100 & -0.0061 & 0.0045 & 0.4722 \end{pmatrix}$	$\begin{pmatrix} 11.6510 & -0.0676 & 0.0651 & -0.5659 \\ -0.0676 & 0.0017 & -0.0019 & -0.0004 \\ 0.0651 & -0.0019 & 0.0056 & 0.0005 \\ -0.5659 & -0.0004 & 0.0005 & 0.0391 \end{pmatrix}$
	**SMELL_PCT**	**HVLT_TOTLEARN**
	$\begin{pmatrix} 9.6941 & -0.0576 & 0.0139 & -0.4583 \\ -0.0576 & 0.0014 & -0.0016 & -0.0003 \\ 0.0139 & -0.0016 & 0.0158 & 0.0005 \\ -0.4583 & -0.0003 & 0.0005 & 0.0314 \end{pmatrix}$	$\begin{pmatrix} 2.6685 & -0.0162 & -0.0028 & -0.1257 \\ -0.0162 & 0.0004 & -0.0004 & -0.0001 \\ -0.0028 & -0.0004 & 0.0117 & 0.0000 \\ -0.1257 & -0.0001 & 0.0000 & 0.0087 \end{pmatrix}$

The cut-off value for the HD-MCI and HD-DM in the kth cognitive domain is given by

$$\text{CUT}_{MCI,k} = \hat{\hat{\mu}}_k - 1.5\sqrt{\hat{V}_k}$$

For an HD gene-expanded individual at age, AGE with the prior number of the kth neuropsychological test, PRACTICE and years of education at study entry, EDUCATION_YEAR, the model-based diagnosis of MCI in the kth cognitive domain will be given if the observed k^{th} neuropsychological test score is below the cut-off value $\text{CUT}_{MCI,k}$.

Additional Data

Table A. The log-logistic AFT model for time to HD motor onset from study entry (baseline). with the predictors of Mild Cognitive Impairment, CAP and TMS at baseline.

Variables	Parameter Estimate	Standard Error	p-value
Inhibition			0.366
Non-MCI	0.073	0.116	
Missing	0.742	0.540	
Working Memory			0.940
Non-MCI	0.031	0.129	
Missing	0.095	0.283	
Motor Planning			0.014
Non-MCI	0.271	0.093	
Missing	0.095	0.225	
Information Integration			0.059
Non-MCI	0.222	0.097	
Missing	-0.218	0.729	
Sensory Processing			0.678
Non-MCI	0.029	0.103	
MIssing	0.357	0.408	
Learning - Memory			0.404
Non-MCI	0.129	0.096	
Missing	0.171	0.863	
TMS	-0.042	0.007	<0.0001
CAP	-0.005	0.001	<0.0001

AFT= Accelerated Failure Time; HD=Huntington’s disease; TMS=Total Motor Score from Unified HD Rating Scale; CAP=CAG repeat length by Age Product.

Table B. The frequency of MCI patterns found during the follow-up for 343 prodromal HD individuals who were cognitively intact at study entry.

MCI Patterns	Frequency
Cognitively intact	180
MCI in single domain	
Inhibition	7
Working Memory	7
Motor Planning	15
Information Integration	18
Sensory processing	38
Learning - Memory	7
Subtotal	92
MCI in two domains	
Inhibition & Motor Planning	2
Inhibition & Information Integration	7
Inhibition & Sensory processing	2
Motor Planning & Information Integration	6
Motor Planning & Sensory processing	4
Motor Planning & Learning - Memory	1
Information Integration & Sensory processing	5
Information Integration & Learning - Memory	1
Sensory processing & Learning - Memory	3
Subtotal	31
MCI in at least three domains	40
Total	343

MCI=Mild Cognitive Impairment; HD=Huntington’s disease
